# Risk stratification and prognostic factors in patients with pulmonary arterial hypertension and comorbidities a cross-sectional cohort study with survival follow-up

**DOI:** 10.1186/s12931-020-01393-1

**Published:** 2020-05-24

**Authors:** Panagiota Xanthouli, Maria Koegler, Alberto M. Marra, Nicola Benjamin, Lukas Fischer, Christina A. Eichstaedt, Satenik Harutyunova, Christian Nagel, Ekkehard Grünig, Benjamin Egenlauf

**Affiliations:** 1 Centre for Pulmonary Hypertension at Thoraxklinik gGmbH at Heidelberg University Hospital, Röntgenstraße 1, 69126 Heidelberg, Germany; 2 Translational Lung Research Centre Heidelberg (TLRC), German Centre for Lung Research (DZL), Heidelberg, Germany; 3 IRCCS SDN, Naples, Italy; 4 Laboratory for Molecular Genetic Diagnostics, Institute of Human Genetics, Heidelberg University, Heidelberg, Germany; 5 Lung Centre, Klinikum Mittelbaden, Baden-Baden Balg, Baden-Baden, Germany

**Keywords:** Pulmonary arterial hypertension, Risk stratification, Comorbidities, Time to clinical worsening, Survival

## Abstract

**Background:**

The objective of this study was to analyze prognostic factors and risk stratification in patients with pulmonary arterial hypertension (PAH) and comorbidities.

**Methods:**

Patients with invasively diagnosed PAH were included in the analysis. Comorbidities were clinically diagnosed as proposed in the 6th World Symposium of pulmonary hypertension. Uni- and multivariate analysis were employed for identification of factors predicting survival and time to first clinical worsening (TTCW). Risk stratification was based on parameters from ESC/ERS-guidelines 2015.

**Results:**

In total 142 patients were enrolled in the study, 90 of them were diagnosed as PAH without and 52 with comorbidities. All patients received targeted PAH therapy and were followed for 3.3 ± 2.4 years. In PAH patients without comorbidities survival and TTCW were significantly associated with reduced 6-min walking distance (6MWD), elevated N-terminal pro brain natriuretic peptide (NT-proBNP), WHO-functional class (WHO-FC) and right atrial (RA) area. In the multivariate analysis, 6MWD was an independent predictor for survival (*p* = 0.002) and WHO-FC for TTCW (*p* = 0.001). In patients with PAH and comorbidities these parameters had no significant association with survival and TTCW. Average risk score was significantly associated with survival (*p* = 0.001) and TTCW (*p* = 0.013) in PAH but not in PAH with comorbidities (both *p* > 0.05; figure 1).

**Conclusion:**

Risk stratification based on ESC/ERS-guidelines could only be confirmed in patients without comorbidities, but not in patients with PAH and comorbidities. The data of this study suggest, that a different risk stratification needs to be applied to PAH patients with comorbidities. Further studies are needed to confirm these results.

**Trial registration:**

Not applicable, retrospective registry.

## Background

In the current guidelines on diagnosis and treatment of pulmonary arterial hypertension (PAH) a risk stratification based approach according to the severity of the disease is recommended for treatment strategy [1]. Current registries selected risk parameters that define a low-, intermediate- and high-risk group and demonstrated the feasibility and validity of this approach [2–8]. According to these and further publications the strategy of risk stratification as basis for treatment decisions has been maintained within the last World Symposium on Pulmonary Hypertension in Nice 2018 [9]. However, previous publications were based on retrospective registry data analyses of patients with “classical” PAH without significant comorbidities as in idiopathic, hereditary PAH and drug/toxin induced PAH [7], or in mixed cohorts of PAH patients both with and without comorbidities [2–4]. Studies focusing on risk stratification in patients with PAH and comorbidities are lacking, although these patients are very common. In Germany, Switzerland and Sweden, the median age of newly diagnosed PAH patients was last reported to be about 65 years, or higher [10–12], whereas it was 50 years or less in recently completed international treatment studies [13–16]. Elderly patients with PAH often present with several comorbidities such as left heart or lung disease [17]. These patients may respond differently to PAH targeted therapies and initial monotherapy might be appropriate [9]. Patients with this condition are often diagnosed at an advanced age (> 75 years) and carry at least three additional risk factors for left heart failure with preserved left ventricular ejection fraction such as high systemic blood pressure, diabetes mellitus, coronary artery disease, atrial fibrillation or obesity [9].

To our knowledge, there is no data available regarding the efficacy of risk stratification parameters among patients with PAH and comorbidities. Therefore, the main objective of this study was to analyze risk stratification parameters recommended in the ESC/ERS-PAH-guidelines in patients with PAH with and without comorbidities to predict survival and/or time to first clinical worsening (TTCW).

## Methods

### Study design

This was a single-center, retrospective study analyzing TTCW and survival during follow-up in PAH-patients diagnosed and treated in the center for PH, Thoraxklinik Heidelberg gGmbH at Heidelberg University Hospital, Germany. TTCW was defined as death, transplantation, hospitalization due to PAH, worsening of functional class of at least one stage and 6-min walking distance (6MWD) deterioration ≥15% compared to baseline. Clinical worsening was recorded either with date and type of worsening or death with date, cause and circumstances. All data were anonymized and the study was approved by the ethics committee of the medical faculty of Heidelberg University Hospital (internal number S-417/2016). The study complied with the Declaration of Helsinki in its current version.

### Study population

From medical records we reviewed all incident (i.e. newly diagnosed) patients aged ≥18 years with PAH (defined according to the ESC/ERS-PH-guidelines) [1] diagnosed and treated at the Thoraxklinik Heidelberg. Inclusion required a baseline right heart catheterisation (RHC) confirming PAH, defined as a mean pulmonary arterial pressure ≥ 25 mmHg, pulmonary arterial wedge pressure ≤ 15 mmHg and pulmonary vascular resistance > 3 Wood units. Patients with significant left heart or lung disease were excluded.

### Definition of PAH and comorbidities

All patients included in the analysis were classified as PAH with regard to hemodynamic criteria according to the current guidelines [1]. PH due to left heart disease was ruled out by volume challenge during RHC by either leg elevation or exercise haemodynamics and under consideration of hydration status, transpulmonary and diastolic pressure gradients. Furthermore, stress echocardiography and left heart catheterization were performed in case of suspected left heart disease. Patients with the diagnosis of PH due to left heart disease according to the guidelines were excluded from the analysis.

PH due to lung disease was ruled out by lung function tests and high-resolution computed tomography. In case of significant lung disease causative of pulmonary vascular abnormalities, patients were excluded from the analysis. Differentiation of PAH with (“atypical PAH”) and without comorbidities (“typical PAH”) was defined according to the criteria from the Cologne consensus conference 2017 [18–20] and from the 6th PH-World Symposium 2018 [21, 22]. Cardiac phenotype was defined as having at least three of the following conditions: systemic arterial hypertension, coronary artery disease, diabetes mellitus, obesity (BMI > 30 kg/m^2^), left atrial enlargement or atrial fibrillation. Pulmonary phenotype was defined as pulmonary disease with normal or near-normal lung function (forced expiratory volume in 1 s (FEV1) ≥ 60% predicted, forced vital capacity (FVC) ≥70% predicted), no clinically significant alterations of lung parenchyma on chest computed tomography (CT), and often present with DLCO < 45% of reference value and hypoxemia. Included PAH patients showed < 20% of pulmonary fibrosis in routinely performed high-resolution chest CT.

### Statistical methods

Statistical analyses were conducted by a medical statistician (NB). Data are described as means ± standard deviations. Survival time was estimated from the first visit till the end of follow-up in this study.

Quantitative characteristics at baseline were compared between PAH patients with and without comorbidities by t-tests with robust variance estimation (Welch-Test). A nonparametric sensitivity analysis (Mann-Whitney test) was performed for baseline variables to test for robustness of results. For comparison of categorical variables between groups chi-square test was used. For analysis of predictive parameters for probability of death and TTCW we performed a univariate and a multivariate analysis.

Metric variables for univariate analysis were selected by clinical significance. Parameters included 6MWD, NT-proBNP, WHO-FC, right atrial (RA) area, right ventricular (RV) area, TAPSE, left ventricular eccentricity index (LV-EI) and RV function (qualitative assessment from normal function to severe impairment during echocardiography and quantitative assessment during RHC).

All variables identified with the univariate logrank tests as being significantly associated with survival (*p* < 0.05) were further analyzed using a multivariate Cox model. Effect sizes are given as hazard ratio point estimates with 95% confidence intervals (CI) within Cox proportional hazard model.

Risk assessment parameters as suggested in ESC/ERS-guidelines were evaluated by uni- and multivariate analysis to compare the groups in terms of survival and TTCW [1]. Accordingly, patients were classified as low, intermediate or high risk for survival or TTCW in the Kaplan-Meier analysis depending on the risk, or average risk in case of risk-sets, for each patient [4, 6]. Different risk stratification sets including the French risk set [2–8], COMPERA [4, 6] and an extended French risk-set including also RA area were used to compare between PAH patients without and with comorbidities. All analyses were performed using IBM SPSS 25 (SPSS Statistics V25, IBM Corporation, Somers, New York).

## Results

### Baseline characteristics

**Fig. 1:**
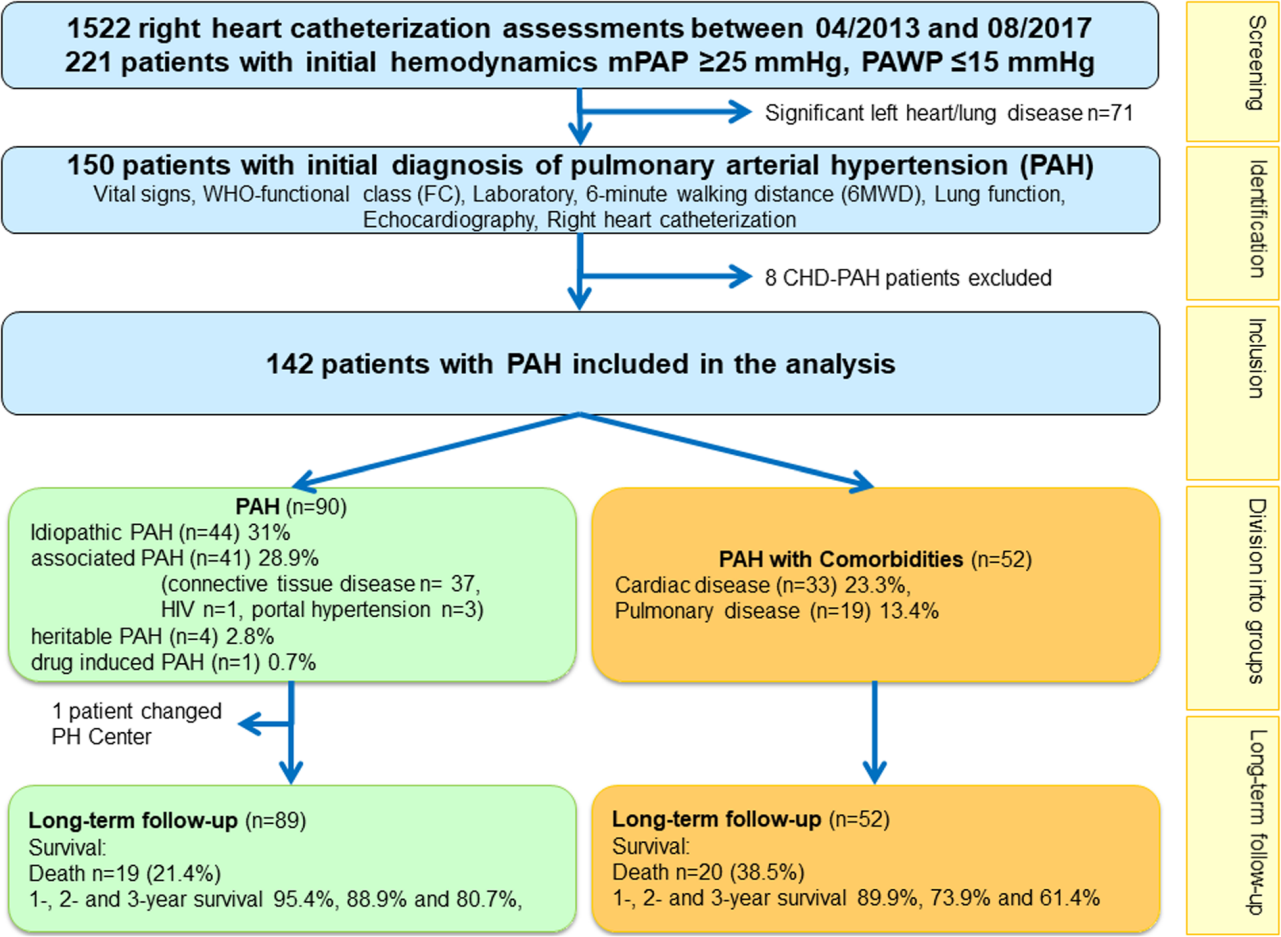
Study flow-chart. Patients were divided according to their PAH phenotype at baseline

Between 04/2013 and 08/2017 1522 RHC assessments were performed in the center for pulmonary hypertension, Thoraxklinik Heidelberg gGmbH at Heidelberg University Hospital. Out of them, 221 incident patients fulfilled the hemodynamic criteria of PAH and were screened for inclusion into the database. Seventy-one out of the 221 patients were excluded due to significant left heart or lung disease. Eight patients with PAH associated with congenital heart disease were excluded. Thus, the final study group consisted of 142 patients with invasively confirmed PAH. These patients were furtherly divided into two groups depending on their comorbidities (Table 1, Fig. 1).

**Table 1:** Demographics, classification and treatment variables

Characteristics	Complete dataset (***n*** = 142)	PAH (***n*** = 90)	PAH with comorbidities (***n*** = 52)	***p***-value
	n (%)	n (%)	n (%)	
Female sex no. [%]	87 (61.3)	66 (73.3%)	21 (40.4%)	< 0.0001
Diagnostic group of PAH [%]				
IPAH	44 (31%)	44 (48.8%)		
HPAH	4 (2.8%)	4 (4.4%)		
DPAH	1 (0.7%)	1 (1.1%)		
APAH	41 (28.9%)	41 (45.6%)		
PAH with cardiac comorbidities	33 (23.3%)		33 (63.5%)	
PAH with pulmonary comorbidities	19 (13.4%)		19 (36.5%)	
Group of APAH [%]				
Connective tissue diseases	37 (26.1%)	37 (41.1%)		
HIV	1 (2.5%)	1 (1.1%)		
Portal hypertension	3 (0.7%)	3 (3.3%)		
No. of PAH drugs				0.82
Monotherapy	90 (63.4%)	56 (62.2%)	34 (65.4%)	
Double combination	50 (35.2%)	33 (36.7%)	17 (32.7%)	
Triple combination	2 (1.4%)	1 (1.1%)	1 (1.9%)	
Type of PAH drug at diagnosis [%]				0.02
Calcium channel blocker	12 (8.5%)	4 (4.4%)	8 (15.4%)	
Endothelin receptor antagonist	62 (43.7%)	48 (53.3%)	14 (26.9%)	
Phosphodiesterase 5 inhibitor	108 (76.1%)	65 (72.2%)	43 (82.7%)	
Prostacyclin	2 (1.4%)	1 (1.1%)	1 (1.9%)	
Soluble guanylate cyclase stimulator	7 (4.9%)	3 (3.3%)	4 (7.7%)	
Oxygen treatment	53 (37.3%)	25 (27.8%)	28 (53.8%)	
Anticoagulants	116 (81.7%)	69 (76.7%)	47 (90.4%)	0.042
Diuretics	108 (76.1%)	63 (70.0%)	45 (86.5%)	0.026

In case of missing data, sample sizes are given in the column of n. sample size is given in case of missing values. *IPAH* idiopathic pulmonary arterial hypertension, *HPAH* heritable pulmonary arterial hypertension, *DPAH* drug-induced pulmonary arterial hypertension, *APAH* associated pulmonary arterial hypertension, *PAH* pulmonary arterial hypertension

Out of 142 PAH patients, 90 had no significant comorbidities (66 females (73%), mean age 59 ± 15.7 years, WHO-FC II 29%, III 63%, IV 8%, 44 patients with idiopathic PAH (31%), 41 patients with associated PAH (28.9%), 4 patients with heritable PAH (2.8%) and 1 patient with drug induced PAH (0.7%).

Fifty-two patients were classified as PAH with comorbidities (33 cardiac, 19 pulmonary phenotype, 40% female (*n* = 21), mean age 70.8 ± 8.7 years, WHO-FC II 6%, III 75%, IV 19%).

In our cohort, patients with systemic sclerosis did not present with significant cardiac or pulmonary comorbidities and were therefore classified as PAH without comorbidities. One patient with cardiac phenotype of PAH presented with Sjögren syndrome. All patients, including patients of the cardiac PAH phenotype, had a good left ventricular function.

PAH patients with comorbidities were significantly more often obese (51.9% vs. 24.4%, *p* = 0.001). Twelve patients (36.4%) with cardiac phenotype presented with more than three comorbidities. PAH patients without significant comorbidities showed no cardiac comorbidity in 31.2% (*n* = 28), one condition in 38.8% (*n* = 35) and 2 conditions in 30% (*n* = 27).

All patients received targeted PAH therapy as monotherapy or combination treatment. The distribution of PAH medication (endothelin receptor antagonists (ERA), phosphodiesterase 5 inhibitors, soluble guanylate cyclase stimulators, oxygen and calcium channel blockers) was comparable between groups (Table 1). Combination of at least two PAH medications was more frequent among PAH patients without comorbidities. PAH patients with comorbidities received monotherapy in 61.5% of cases. Patients with comorbidities received significantly more often anticoagulants (*p* = 0.042) and diuretics (*p* = 0.026). Both groups did not significantly differ concerning their hemodynamic values measured by RHC at baseline (Table 2). PAH patients with comorbidities at baseline had lower exercise capacity with 6MWD (271.8 ± 122.3 m vs. 372.6 ± 115.5 m, *p* < 0.0001), lower DLCO (40 ± 19.4% predicted vs. 53.5 ± 21.7% predicted, *p* = 0.001), lower glomerular filtration rate (55.95 ± 18.5 pg/ml vs. 77.4 ± 25.0 pg/ml, *p* < 0.0001), larger RA area (22.4 ± 5.9 cm^2^ vs. 19.2 ± 6.3 cm^2^, *p* = 0.003) and were more frequently in worse WHO-FC (Table 2, *p* = 0.021) compared to those without comorbidities. Except for NT-proBNP (nonparametric *p* = 0.046), results were confirmed by the nonparametric sensitivity analysis.

**Table 2:** Clinical characteristics at baseline

Characteristics	Complete dataset (***n*** = 142)		PAH (***n*** = 90)		PAH with comorbidities (***n*** = 52)		***p***-value between groups
	Mean ± SD or n (%)	n	Mean ± SD or n (%)	n	Mean ± SD or n (%)	n	
Age [years]	63.3 ± 14.7		59.0 ± 15.7		70.8 ± 8.7		< 0.0001
Vital signs							
Heart rate [/min]	79 ± 14		79 ± 13		78 ± 12		0.70
Oxygen saturation SaO_2_ [%]	94.7 ± 2.7	106	95.6 ± 2.4	62	93.6 ± 3.3	44	0.001
WHO-FC no. [%]							0.021
II	25 (20.2%)		22 (29.0%)		3 (6.3%)		
III	84 (67.7%)		48 (63.2%)		36 (75.0%)		
IV	15 (12.1%)		6 (7.8%)		9 (18.7%)		
Laboratory							
NT-proBNP [ng/l]	2334 ± 3270	104	2063 ± 3427	65	2786 ± 2976	39	0.26
Creatinine [mg/dl]	1.04 ± 0.42	141	0.93 ± 0.39	89	1.24 ± 0.4	52	< 0.0001
GFR [ml/min/1.73m^2^]	69.52 ± 24.99	136	77.41 ± 24.96	86	55.95 ± 18.52	50	< 0.0001
Ferritin [μg/l]	194.8 ± 188.5	77	177.7 ± 151	47	221.4 ± 236.1	30	0.33
6MWT							
6MWD [m]	332.6 ± 127.7	111	372.6 ± 115.5	67	271.8 ± 122.3	44	< 0.0001
Lung function tests							
DLCOsb [%]	48.4 ± 21.8	124	53.5 ± 21.7	77	40.0 ± 19.4	47	0.001
TLC [%]	90.5 ± 20.5	136	90.2 ± 21.1	85	90.9 ± 19.7	51	0.85
FEV1 [%]	83.9 ± 23.2	138	83.2 ± 26.0	86	85.0 ± 17.7		0.62
Right heart catheterization at rest							
RAP [mmHg]	7.9 ± 4.8	104	7.1 ± 4.4	63	9.2 ± 5.2	41	0.033
mPAP [mmHg]	43.2 ± 11.7		43.1 ± 12.0		43.4 ± 11.2		0.88
Cardiac output [l/min]	4.6 ± 1.2	126	4.6 ± 1.3	79	4.4 ± 1.1	47	0.36
Cardiac index [l/min/m^2^]	2.4 ± 0.6	117	2.5 ± 0.6	72	2.3 ± 0.5	44	0.035
PAWP [mmHg]	9.6 ± 3.1	136	9.3 ± 3.2	84	10.2 ± 2.9		0.084
PVR [dyn*s*cm^−5^]	648 ± 326	135	655 ± 351	84	635 ± 284	51	0.71
SvO_2_ [%]	65.5 ± 8.6	95	66.0 ± 9.3	58	62.0 ± 6.8	37	0.020
Echocardiography							
RA [cm^2^]	20.4 ± 6.3	136	19.2 ± 6.3	85	22.4 ± 5.9	51	0.003
RV [cm^2^]	24.2 ± 7.0	138	23.5 ± 7.2	87	25.4 ± 6.6	51	0.13
sPAP [mmHg]	63.5 ± 19.5	140	62.6 ± 20.11	88	65.2 ± 18.4	52	0.43
TAPSE [cm]	1.9 ± 0.5	139	1.97 ± 0.53	88	1.90 ± 0.56	51	0.47
LV-EI	1.28 ± 0.25	113	1.3 ± 0.2	74	1.3 ± 0.3	39	0.85
RV pump function no. [%]							
normal	22 (15.8%)		19 (21.8%)		3 (5.9%)		
mild impairment	15 (10.8%)		7 (8.0%)		8 (15.7%)		
moderate impairment	28 (20.1%)		15 (17.2%)		13 (25.5%)		
severe impairment	74 (53.3%)		46 (53.0%)		28 (52.9%)		

*SaO*_*2*_ oxygen saturation, *WHO-FC* World Health Organization Functional Class, *NT-proBNP* n-terminal pro brain natriuretic peptide, *GFR* glomerular filtration rate, *6MWT/D* 6-min walking test/distance, *DLCOsb* diffusion capacity of the lung single breath, *TLC* total lung capacity, *FEV1* forced expiratory volume in one second, *RAP* right atrial pressure, *mPAP* mean pulmonary arterial pressure, *PAWP* pulmonary arterial wedge pressure, *PVR* pulmonary vascular resistance, *SvO*_*2*_ mixed venous oxygen saturation, *RA* right atrial, *RV* right ventricular, *sPAP* systolic pulmonary arterial pressure, *TAPSE* tricuspid annular plane systolic excursion, *LV-EI* left ventricular eccentricity index

### Survival and clinical worsening

In the observation period, 39 patients died (27.7%): 19 PAH patients without comorbidities (21.4%; 1-, 2- and 3-year survival 95.4, 88.9 and 80.7%, respectively) compared to 20 PAH patients (38.5%; 1-, 2- and 3-year survival 89.9, 73.9 and 61.4%, respectively) with comorbidities.

First clinical worsening was documented as hospitalisation in the follow-up time due to PAH in 48 patients, 26 events in the PAH group without and 22 events in the PAH group with comorbidities. Worsening of symptoms was present in 19 patients, 13 in the classical PAH group and six among the PAH group with comorbidities. Mean time to clinical worsening was 4.67 ± 0.44 years for PAH without comorbidities and 3.45 ± 0.54 years for PAH with comorbidities.

### Prognostic impact of baseline parameters

Among PAH patients without comorbidities 6MWD, NT-proBNP and RV pump function significantly correlated with survival and TTCW (Table 3). In the univariate Cox regression analysis of the whole cohort baseline 6MWD (*p* < 0.0001 survival and TTCW), NT-proBNP (*p* = 0.006 survival, *p* = 0.024 TTCW), WHO-FC (*p* = 0.005 survival, *p* = 0.001 TTCW), TAPSE (*p* = 0.006 survival, *p* = 0.049 TTCW), and worse RV pump function (*p* < 0.0001 survival and TTCW) were significantly associated with survival and TTCW. Echocardiographic parameters of right heart size (RA and RV area) were additionally identified (*p* = 0.002 and *p* = 0.017, respectively) in the univariate analysis as predictors for TTCW (Table 3).

**Table 3:** Cox regression of baseline values in uni- and multivariate (*) analysis

Baseline values	Whole cohort		PAH		PAH with comorbidities	
	Cox regression	n	Cox regression	n	Cox regression	n
	*p*-value		*p*-value		*p*-value	
**Survival**						
6MWD	**< 0.0001**	137	**0.006**	65	0.052	42
NT-proBNP	**0.006**	102	**0.011**	63	0.356	37
WHO-FC	0.005*	121	0.001*	74	0.403	46
RA area	0.254	133	0.991	82	0.472	49
RV area	0.778	135	0.631	84	0.505	49
TAPSE	**0.006**	137	**0.033**	86	0.220	49
LV-EI	0.613	111	0.896	72	0.282	38
RV pump function	**< 0.0001**	109	**< 0.0001**	85	0.499	49
**Time to clinical worsening**						
6MWD	< 0.0001*	109	**0.02**	65	0.047*	44
NT-proBNP	**0.024**	102	0.07	63	0.485	39
WHO-FC	**0.001**	122	< 0.0001*	74	0.346	48
RA area	**0.002**	133	0.067	82	0.074	51
RV area	**0.017**	135	0.073	84	0.342	51
TAPSE	**0.049**	137	0.071	86	0.679	51
LV-EI	0.096	111	0.398	72	0.065	39
RV pump function	< 0.0001*	135	**< 0.0001**	84	0.344	51

*6MWD* 6-min walking distance, *NT-proBNP*: n-terminal pro brain natriuretic peptide, *WHO-FC* World Health Organization Functional Class, *RA* right atrial, *RV* right ventricular, *TAPSE* tricuspid annular plane systolic excursion, *LV-EI* left ventricular eccentricity index. Parameters that were used for multivariate analysis are written in bold. *Denotes significant parameters in multivariate analysis

In the multivariate analysis WHO-FC was the only independent predictor of survival and RV pump function and 6WMD for TTCW (Table 3).

Among PAH patients with comorbidities, 6MWD at baseline was significantly associated with TTCW (*p* = 0.047) and showed a tendency to predict survival (*p* = 0.052). None of the other values were found to be associated with survival and TTCW in this group.

### ESC/ERS risk stratification – mean risk categories

Three risk categories (low, intermediate and high risk) were derived from non-invasive parameters of the ESC/ERS risk score including 6MWD, NT-proBNP, WHO-FC and RA area. The patient allocation to the three risk categories corresponded to significantly different survival and TTCW in the whole cohort for all parameters. Risk stratification of patients with PAH without comorbidities showed also significant differences for the three risk groups for almost all parameters (except for WHO-FC, which showed a tendency for prediction of TTCW), while risk of PAH with comorbidities (Table 4) did not significantly differ between the three risk groups for all parameters.

**Table 4:** ESC/ERS risk score 2015 assessments included

ESC/ERS risk score groups	Whole cohort		PAH		PAH with comorbidities	
Baseline values	Kaplan-Meier *p*-value	n	Kaplan-Meier *p*-value	n	Kaplan-Meier *p*-value	n
**Survival**						
6MWD	**0.010**	113	**0.019**	68	0.636	45
NT-proBNP	**0.031**	104	**0.032**	64	0.260	40
WHO functional class	**0.004**	126	**0.016**	77	0.491	49
Right atrial area	**0.045**	135	**0.013**	84	0.774	51
**Time to first clinical worsening event**						
6MWD	**0.011**	112	**0.031**	67	0.129	45
NT-proBNP	**0.012**	103	**0.002**	63	0.194	40
WHO functional class	**0.006**	125	0.079	76	0.547	49
Right atrial area	**0.001**	134	**0.034**	83	0.244	51

*6MWD* 6-min walking distance, *NT-proBNP* N-terminal pro brain natriuretic peptide, *WHO* World Health Organization

**Fig. 2:**
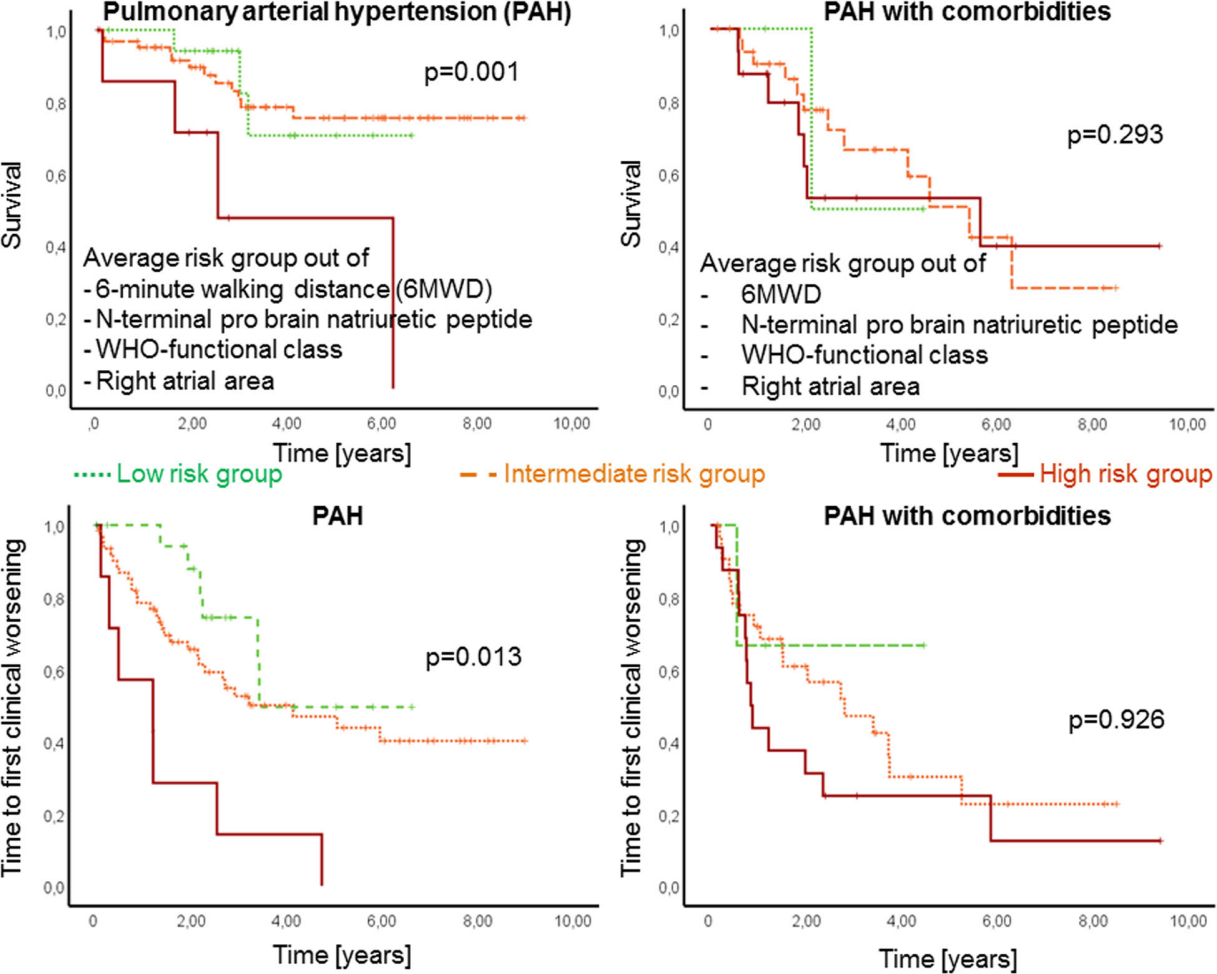
Kaplan Meier Curves. Survival and time to clinical worsening curves of patients with PAH (left) and PAH with comorbidities (right) in the average risk group of four risk factors. Only PAH patients without comorbidities showed significant differences in survival and time to clinical worsening in average risk groups

When using an average value of the four ESC/ERS risk parameters (6MWD, WHO-FC, NT-proBNP, RA area) to divide patients into three risk groups, Kaplan-Meier analysis showed significantly different survival (*p* = 0.001) and TTCW (*p* = 0.013) among PAH patients without comorbidities, but not among PAH with comorbidities (*p* = 0.293 and *p* = 0.926, respectively; Fig. 2).

The average risk group from the non-invasive French-modified ESC/ERS risk-set (6MWD, WHO-FC, NT-proBNP) showed significant prediction of survival for PAH without comorbidities (*p* = 0.002), but not for PAH with comorbidities (*p* = 0.766). For TTCW, risk stratification according to the French risk-set showed significant prediction for PAH without comorbidities (*p* = 0.001), but not for PAH with comorbidities (*p* = 0.837). By use of the COMPERA risk score (6MWD, WHO-FC, NT-proBNP, RA pressure, cardiac index and mixed oxygen saturation), survival and TTCW were significantly stratified for PAH without (*p* = 0.003 and *p* = 0.038), but not with comorbidities (*p* = 0.435 and *p* = 0.637).

## Discussion

This is the first study comparing risk stratification parameters in PAH patients with and without cardio-pulmonary comorbidities. Risk stratification, based on ESC/ERS risk calculation parameters, was able to predict survival and TTCW only among patients with PAH without comorbidities, but not in PAH with comorbidities. In comorbid PAH patients, only 6MWD had a predictive value for TTCW.

While the role of PAH among patients with comorbid conditions was reevaluated during the 6th World Symposium of PH in Nice, a separate risk stratification strategy has not yet been implemented for this particular group [21]. The data of this study suggest that a different risk stratification needs to be applied to PAH patients with comorbidities. Further prospective studies are needed to confirm these results.

### Predictive power of echocardiographic assessments

In our cohort, TAPSE, as an indicator of RV-dysfunction, was predictive of survival for the whole cohort and PAH without comorbidities. For TTCW, TAPSE was predictive for the whole cohort and in trend for PAH without comorbidities. This finding is in line with the predictive power of RV function as qualitative parameter [23], which showed also significant prediction for TTCW in PAH without comorbidities in our cohort. Both RA and RV areas showed only predictive power for the whole cohort, and for PAH without comorbidities in trend. This might be due to an acute volume overload, which led to clinical worsening and hospitalization shortly after diagnosis, but did not impair survival.

### Risk stratification in PAH with comorbidities

In the COMPERA registry, patients were enrolled regardless their comorbidity status and the mortality rate was reported to be 30.3% within 5 years [24]. In our cohort the overall mortality was 27.7%, similar to the finding of the COMPERA-analysis [24]. Our findings are in line with previously published evaluations of risk stratification tools showing significant results for mixed cohorts of PAH patients with and without comorbidities [2–4]. There is contradicting evidence on survival in patients with PAH and comorbidities. Our findings are in contrast to the study from Opitz et al. 2016, who reported similar survival between patients with IPAH and atypical PAH [17]. However, our study is in line with findings of Hjalmarsson et al. 2018 reporting impaired survival in PAH patients with comorbidities, in particular ischemic heart disease and renal dysfunction [25]. Furthermore, patients with PAH and diabetes mellitus have shown significantly lower 10-year survival [26].

While known risk parameters for PAH significantly stratified survival and TTCW in PAH without comorbidities, only 6MWD was associated with TTCW in PAH with comorbidities in our cohort. 6MWD has also been described as independent prognostic predictor both in chronic left heart failure [27] and in chronic obstructive pulmonary disease [28] representing a common stratification parameter for PAH, cardiac and pulmonary phenotypes.

Surprisingly, all other clinical parameters such as WHO-FC and echocardiographic parameters were not predictors of survival or TTCW in our PAH patients with comorbidities. Co-existing lung or left heart disease can affect the interpretation of exercise capacity and WHO-FC of patients with PAH [29].

### Medical treatment and risk stratification for PAH with comorbidities are unclear

Almost all randomized controlled trials of PAH medication have included predominantly younger patients with “classic” PAH without comorbidities. The phenotypes of PAH with comorbidities have not yet been fully characterized and controlled drug trials are missing. In PAH with comorbidities the treatment algorithm is less clear, monotherapy has been recommended in comorbid PAH patients [9]. A recently published post-hoc analysis of the AMBITION trial showed that patients with PAH and cardiovascular risk factors displayed an attenuated response to double combination therapy [30] and tended to discontinue the therapy with ERA due to lack of tolerance of the medication and lack of efficacy. Our cohort also showed a higher amount of monotherapy (61.5%) in PAH patients with comorbidities than double or triple therapy (34.5 and 3.8%, respectively) compared to PAH patients without comorbidities. These observations show that the evidence to guide medical treatment by risk stratification is at least lower in elderly patients with PAH and comorbidities.

### Study limitations

Due to the retrospective nature of the study, selection bias may not be excluded. However, patients’ records were derived from the RHC data laboratory, including all available cases of initial diagnosis at our centre.

Results may also be influenced by the small sample size of the study, especially regarding PAH patients with comorbidities. However, results were consistent for almost all stratification parameters and significant prediction of TTCW was also detected in a sample of 44 PAH patients with comorbidities. Strata for the average risk score of the four ESC/ERS risk parameters showed similar survival rates for the low and intermediate risk group. Further studies with larger sample sizes are needed to investigate, whether the overlap occurred due to small sample size, or is due to a similar survival rate.

Our analysis of predictive factors was limited to non-invasive risk parameters, as these are routinely performed and commonly available in patients with PAH. Risk stratification in PAH with comorbidities needs to be evaluated in invasive parameter sets and follow-up examination results in future studies.

## Conclusion

Risk stratification based on ESC/ERS-guidelines 2015 could only be confirmed in patients without comorbidities, but not in patients with PAH with comorbidities. The data of this study suggest that different risk stratification and treatment recommendations need to be applied to PAH patients with comorbidities matched to age and concomitant diseases. Further studies are needed to confirm these results and to broaden the knowledge on this PAH phenotype.

## Data Availability

The datasets used and/or analysed during the current study are available from the corresponding author on reasonable request.

## Abbreviations

6MWD: 6-min walking distance
APAH: Associated pulmonary arterial hypertension
CT: Computed tomography
DLCO: Diffusion capacity of carbon monoxide
ERA: Endothelin receptor antagonist
ERS: European Respiratory Society
ESC: European Society of Cardiology
HIV: Human immunodeficiency virus
LV-EI: Left ventricular eccentricity index
NT-proBNP: N-terminal pro brain natriuretic peptide
PAH: Pulmonary arterial hypertension
RA: Right atrial
RHC: Right heart catheterization
RV: Right ventricular
TAPSE: Tricuspid annular plane systolic excursion
TTCW: Time to clinical worsening
WHO-FC: World Health Organization Functional Class
